# Impact of oxygen and glucose availability on the viability and connectivity of islet cells: A computational study of reconstructed avascular human islets

**DOI:** 10.1371/journal.pcbi.1012357

**Published:** 2024-08-13

**Authors:** Gerardo J. Félix-Martínez, Diana Osorio-Londoño, J. Rafael Godínez-Fernández

**Affiliations:** 1 Investigadoras e investigadores por México, Consejo Nacional de Humanidades, Ciencias y Tecnologías, México City, México; 2 Department of Electrical Engineering, Universidad Autónoma Metropolitana, Iztapalapa, México City, México; University of Michigan, UNITED STATES OF AMERICA

## Abstract

The experimental study and transplantation of pancreatic islets requires their isolation from the surrounding tissue, and therefore, from the vasculature. Under these conditions, avascular islets rely on the diffusion of peripheral oxygen and nutrients to comply with the requirements of islet cells while responding to changes in body glucose. As a complement to the experimental work, computational models have been widely used to estimate how avascular islets would be affected by the hypoxic conditions found both in culture and transplant sites. However, previous models have been based on simplified representations of pancreatic islets which has limited the reach of the simulations performed. Aiming to contribute with a more realistic model of avascular human islets, in this work we used architectures of human islets reconstructed from experimental data to simulate the availability of oxygen for *α*, *β* and *δ*-cells, emulating culture and transplant conditions at different glucose concentrations. The modeling approach proposed allowed us to quantitatively estimate how the loss of cells due to severe hypoxia would impact interactions between islet cells, ultimately segregating the islet into disconnected subnetworks. According to the simulations performed, islet encapsulation, by reducing the oxygen available within the islets, could severely compromise cell viability. Moreover, our model suggests that even without encapsulation, only microislets composed of less than 100 cells would remain viable in oxygenation conditions found in transplant sites. Overall, in this article we delineate a novel modeling methodology to simulate detailed avascular islets in experimental and transplant conditions with potential applications in the field of islet encapsulation.

## Author summary

Pancreatic islets are clusters of cells dispersed throughout the pancreas that play a critical role for the regulation of blood glucose in the human body. Islets are mainly composed of *α*, *β* and *δ*-cells that respectively secrete glucagon, insulin and somatostatin in response to glucose changes. When islets are isolated, either to use them for transplantation for the treatment of type 1 diabetes or to study them experimentally, they are removed from the vasculature, thus limiting the oxygenation and nutrition of islet cells. Moreover, aiming to protect transplanted islets from the immune system, encapsulation of isolated islets is intensively explored. Under these conditions, isolated islets, either naked or encapsulated, rely entirely on the diffusion of oxygen and nutrients available in their surroundings until a new functional vasculature is developed. In this work, we use a detailed computational model of avascular islets based on reconstructed architectures of human islets to study how the availability of oxygen and glucose impacts the viability and connectivity of islet cells emulating experimental and transplant conditions.

## Introduction

Pancreatic islets are endocrine microorgans composed of hundreds or thousands of cells [1], key for the tight regulation of blood glucose. Islets are mainly composed of three cell populations: insulin-producing *β*-cells, glucagon-producing *α*-cells and somatostatin-producing *δ*-cells, which together account for the great majority of islet cells [2]. In normal physiological conditions, insulin is secreted in response to an increase in glucose concentration to promote the uptake of glucose in liver, muscle and adipose tissue. In contrast, glucagon is secreted when glucose is low to promote the release of stored glucose to restore blood glucose to normal levels. Somatostatin, on the other hand, participates indirectly by inhibiting the secretion of both glucagon and insulin [3]. Regulation of blood glucose requires not only the secretion of insulin, glucagon and somatostatin, but also the tight coordination of islet cells via a myriad of cell-to-cell interactions, including direct electrical communication and paracrine signals between the three types of cells [4–6], which heavily depend on the islet architecture; that is, the number, organization and distribution of *α*, *β* and *δ*-cells. In fact, it has been shown that coordinated secretion of insulin, glucagon and somatostatin is lost as a consequence of pathological disturbances such as diabetes [7–9].

In normal conditions in the human body, islets receive about 20% of blood flow despite the fact that islets mass only accounts for 1–2% of total pancreas mass [10]. Accordingly, in physiological conditions, pancreatic islets are densely vascularized [11–14]. Moreover, the close location between islet cells and blood vessels ensures a swift secretory response to the dynamic glucose changes [15, 16]. These facts highlight the importance of blood supply for the adequate oxygenation and nutrient availability for islet cells, as well as enabling a rapid response to changes in glucose levels.

The current clinical treatment for T1D, an autoimmune disease where *β*-cells are lost [17], is the artificial application of exogenous insulin, either through injections or insulin pumps. However, this treatment has important limitations including the ineffective regulation of insulin and glucose levels and the potential risk of lethal hypoglycemic events [18]. For this reason, islet transplantation, in combination with an immunosuppression scheme, has been proposed as an alternative therapeutic strategy for the treatment of T1D [19, 20], although its application remains limited due to obstacles such as the amount of donor tissue required, the limited availability of oxygen and nutrients at transplant sites, the risk of infection, side effects and possible graft failure due to immune rejection [21]. Encapsulation of isolated islets has been vastly explored as a potential strategy for the immunosuppression-free therapy for T1D [22, 23] using a biocompatible material that affords the diffusion of secreted hormones, nutrients and oxygen while protecting the islets from the immune system. However, this is still a current research field with many challenges, such as biocompatibility issues, nutrition, oxygenation and revascularization of transplanted islets which varies depending on the implantation site [24].

*In vitro* studies of pancreatic islets, as well as islet transplantation, either naked or encapsulated, involve their isolation from the surrounding tissue. As a consequence, islets become avascular tissue disconnected from the blood stream, their primary source of nutrients and oxygen. After transplantation, islets revascularize within 10–14 days, although some reports indicate that it could take longer [25], and in some cases, that the density of the developed vasculature is considerably lower than that of native islets [26]. Under these conditions, passive diffusion becomes the only means of oxygenation for islet cells [27–29]. As a consequence, islet functionality depends on the availability of peripheral oxygen, determined by the culture conditions for *in vitro* experiments or the conditions of the transplant site [30]. Although the liver has been the preferred site for islet transplantation, many associated complications have been identified such as inflammatory reactions, thrombosis and delayed revascularization [31–33]. To prevent these complications that can compromise the availability of oxygen and nutrients, optimal transplantation sites affording high vascularization are still under investigation to enhance functionality and limit graft failure [34]. Potential transplant sites include muscle tissue [35], bone marrow [36], the omentum [37] and the subcutaneous space [38, 39], among others, each of which having its own limitations in terms of surgical availability, allowed graft volume, immune reaction, vascularization and oxygenation. [32, 34].

Computational models have been widely used to evaluate the availability of oxygen and nutrients for avascular islets, emulating both *in vitro* and transplant conditions [30, 33, 40–45], giving a valuable insight into the optimal conditions for islet isolation, culture and transplantation. However, in spite of these advances, models proposed so far have lacked key morphological aspects such as realistic islet architectures (i.e. cell composition, distribution, and organization of *α*, *β* and *δ*-cells). Furthermore, earlier models have overlooked the fact that different cell types consume oxygen and take up glucose at varying rates depending on the glucose concentration. Based on this, in this work we developed a three-dimensional model of islet oxygenation aiming to provide a quantitative description of the viability of avascular human islets with details at the cellular level emulating experimental and transplant conditions.

## Materials and methods

### General methodology

**Fig 1:**
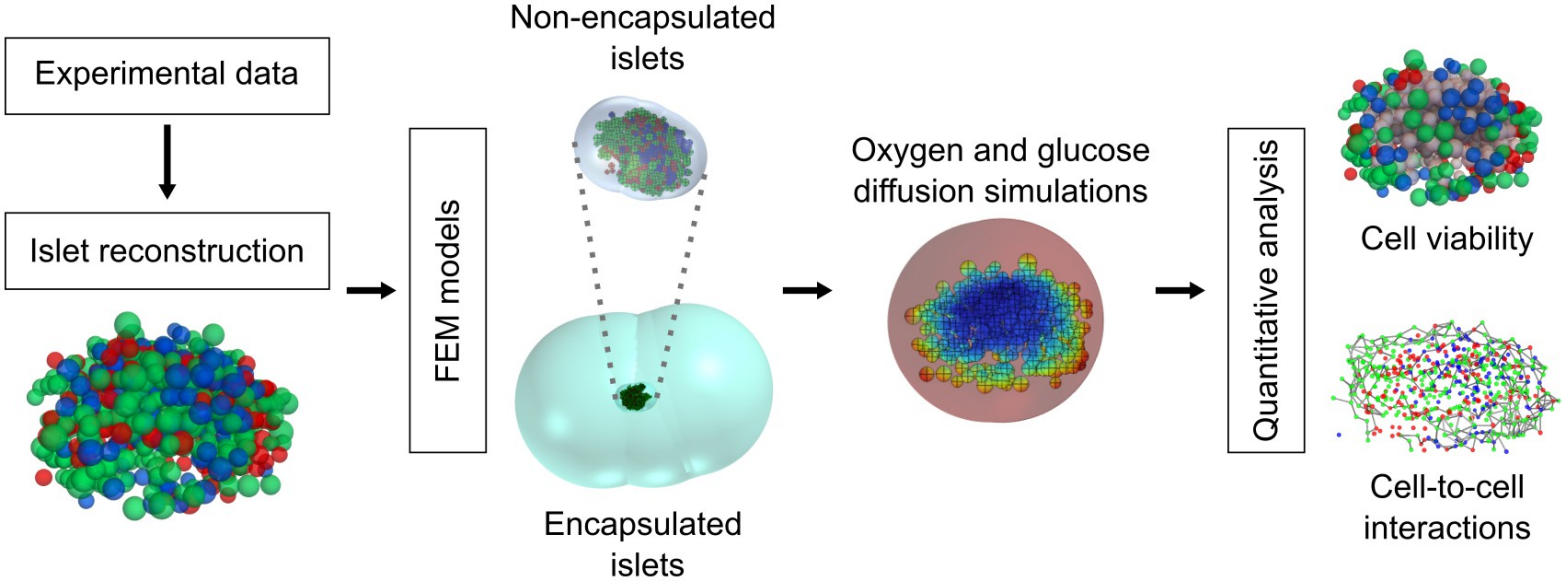
General methodology. Human islets were computationally reconstructed based on experimental data. Models of avascular islets, naked and encapsulated, were built using the finite element method (FEM) to simulate the diffusion of oxygen and glucose throughout the islets. The simulation results were used to perform a quantitative analysis of the viability of islet cells and the impact of non-viable cells on cell-to-cell interactions considering the morphological details of actual human islets.

The modeling methodology used in this work is summarized in Fig 1. Firstly, based on experimental data, human islets of different sizes and shapes were computationally reconstructed. Then, the reconstructed islets were used to implement three-dimensional models of the diffusion of oxygen and glucose throughout avascular islets, both naked and encapsulated, using the finite element method (FEM). Afterwards, we performed a quantitative analysis to determine the oxygenation state (i.e. non-viable, hypoxic or functional) of each *α*, *β* and *δ*-cell. Finally, we evaluated the impact of non-viable cells on cell-to-cell interactions and estimated the optimal islet size depending on the oxygenation of potential transplant sites.

### Reconstruction of human pancreatic islets

Six human islets were reconstructed based on the islet architectures kindly shared by Hoang et al. [46] to the research community, who used confocal microscopy and immunofluorescence techniques to determine the position and identity of *α*, *β* and *δ*-cells in six human islets. Using this information, islet architectures were reconstructed in IsletLab [47] using computational optimization as previously reported [48]. To summarize, the reconstruction algorithm proposes an initial islet by positioning spherical cells at the experimental nuclei coordinates, with randomly assigned radii selected from experimentally-derived distributions. Then, the number of overlapped cells is calculated from the initial islet architecture. In each iteration, during the optimization process, a cell is randomly selected for which new center coordinates and radius are proposed. The number of overlapped cells is calculated in each iteration and compared to the minimum value obtained so far. If the number of overlapped cells is reduced, the proposed changes are accepted. Otherwise, the changes may be accepted or rejected based on a decreasing probability function, in order to prevent the algorithm from becoming trapped in a local minimum. This iterative process continues until convergence or a stopping criterion is met. The interested reader can find a complete description of the reconstruction process in Ref [48]. The characteristics of the reconstructed human islets are given in Table 1.

**Table 1:** Number of cells in the reconstructed human islets.

Islet	*N*_*αβδ*_ (%)	*N*_*α*_ (%)	*N*_*β*_ (%)	*N*_*δ*_ (%)
1	583 (100)	148 (25.4)	316 (54.2)	119 (20.4)
2	2252 (100)	427 (19.0)	1461 (64.9)	364 (16.2)
3	3223 (100)	1082 (33.6)	1524 (47.3)	617 (19.1)
4	3516 (100)	961 (27.3)	2209 (62.8)	346 (9.8)
5	2084 (100)	642 (30.8)	1168 (56.0)	274 (13.1)
6	2841 (100)	830 (29.2)	1355 (47.7)	656 (23.1)

### Modeling the diffusion of oxygen and glucose throughout the reconstructed islets

The diffusion of oxygen and glucose throughout the reconstructed avascular human islets was simulated using the finite element method (FEM) in COMSOL Multiphysics (COMSOL AB, Stockholm, Sweden). The islet boundary was generated programmatically based on the reconstructed islet architecture. Reconstructed islets were meshed using the predefined “Extremely Fine” physics-controlled meshing sequence using tetrahedral elements with minimum and maximum sizes of 0.08 ± 0.02 *μ*m and 8.02 ± 1.95 *μ*m, respectively. The diffusion of oxygen and glucose through the intercellular space was simulated by solving the three-dimensional steady state diffusion equations:

$$\begin{array}{c} D_{O_{2}}^{t}\nabla^{2}\left[O_{2}\right]=0 \end{array}$$ (1)

$$\begin{array}{c} D_{G}^{t}\nabla^{2}\left[G\right]=0 \end{array}$$ (2)

where [*O*_2_] and [*G*] are the concentrations of oxygen and glucose respectively and $D_{O_{2}}^{t}=2.1\times 10^{-9}m^{2}/s$ and $D_{G}^{t}=0.3\times^{-9}m^{2}/s$ are the corresponding diffusion coefficients in the islet tissue [30, 41, 49]. Oxygen tension (*pO*_2_) was obtained assuming an oxygen solubility ($\alpha_{O_{2}}$) in the islet of 2.34 × 10^−4^ mol/(mmHg ⋅ m^3^) (0.17 mol/(atm ⋅ m^3^)) [30]. The boundary conditions adopted involved maintaining constant oxygen and glucose concentrations at the islet periphery, utilizing oxygen partial pressures within the range measured at potential implant sites and *in vitro* experiments (see Table 2) and glucose concentrations commonly used to test the functionality of pancreatic islets (1, 6 and 20 mM) [50]. Throughout the article, these glucose levels are referred to as G_1_, G_6_ and G_20_, respectively (*G*_*i*_ in Eq 4 below). Mathematically, oxygen and glucose concentrations at the islet boundary ([*O*_2_]_*b*_ and [*G*]_*b*_, respectively) were expressed as:

$$\begin{array}{c} {\left[O_{2}\right]}_{b}=\alpha_{O_{2}}pO_{2} \end{array}$$ (3)

$$\begin{array}{c} {\left[G\right]}_{b}=G_{i}. \end{array}$$ (4)

**Table 2:** Oxygen tensions (*pO*_2_) emulating potential transplant sites and *in vitro* culture conditions. Concentrations were obtained as $\left[O_{2}\right]=\alpha_{O_{2}}pO_{2}$, where $\alpha_{O_{2}}$ is the oxygen solubility in the islet of 2.34×10^−4^ mol/(mmHg ⋅ m^3^) as reported by Komatsu et al. [30].

*pO*_2_ **(mmHg)**	[*O*_2_] (*μ*M)	**Transplant site**	**Ref.**
10	2.24	Superficial skin	[51–53]
30	6.71	Dermis, subdermis, intramuscular	[51, 52, 54, 55]
50	11.18	Intestine, intrahepatic, bone marrow	[52, 56–58]
70	15.66	Renal cortex	[59]
*pO*_2_ **(mmHg)**	[*O*_2_] (*μ*M)	**Culture** O_2_	**Ref.**
100	22.37	10%	[60]
160	35.79	21%	[60]
270	60.39	35%	[60]
350	78.29	50%	[60]

Initially, the concentrations of both oxygen and glucose were set to zero in all the geometric domains. Three oxygen ranges were defined following previous works [41, 49, 51] to identify the islet cells as functional ($\bar{pO_{2}}\geq 10\;\text{mmHg}$), hypoxic ($0.45\;\text{mmHg}\;<\bar{pO_{2}}<10\;\text{mmHg}$) and non-viable ($\bar{pO_{2}}<0.45\;\text{mmHg}$), determined by the average surface oxygen ($\bar{pO_{2}}$) at the cell membranes. Negative surface fluxes of oxygen and glucose were adopted as boundary conditions at the cells’ boundaries to represent oxygen consumption and glucose uptake as:

$$\begin{array}{c} -n\cdot J_{ocr\vee gcr}^{i,\sigma }=\{\begin{array}{cc} 0 & \bar{pO_{2}}<0.45\;\text{mmHg}\\ \frac{J_{ocr\vee gcr}^{\sigma }}{A_{i}} & \bar{pO_{2}}\geq 0.45\;\text{mmHg} \end{array} \end{array}$$ (5)

where ***n*** is the unitary vector normal to the cell surface, $J_{ocr\vee gcr}^{i,\sigma }$ indicates the consumption rates of oxygen ($J_{ocr}^{\sigma }$) and glucose ($J_{gcr}^{\sigma }$) by cell *i* of type *σ* (*α*, *β* or *δ*), and *A*_*i*_ is the surface area of cell *i* obtained from the reconstruction process. As expressed in Eq 5, cells identified as non-viable were assumed to stop consuming both oxygen and glucose. For a visual representation of the models, including boundary conditions for both naked and encapsulated islets, see the schematic diagrams provided in S1 and S2 Figs. Values used for the oxygen and glucose consumption rates are described in the following sections and given in Table 3.

**Table 3:** Oxygen and glucose consumption rates for *α*, *β* and *δ*-cells at 1, 6 and 20 mM glucose. Glucose and oxygen consumption rates are denoted as $J_{gcr}^{\sigma }$ and $J_{ocr}^{\sigma }$, respectively, with *σ* indicating the type of cell (*α*, *β* or *δ*).

	Glucose (mM)		
	1	6	20
$J_{gcr}^{\alpha }$ (×10^−6^ mol/s)	8.53	3.14	5.6
$J_{gcr}^{\beta /\delta }$ (×10^−6^ mol/s)	2.54	4.24	9.0
$J_{ocr}^{\alpha }$ (×10^−16^ mol/s)	2.06	0.78	1.2
$J_{ocr}^{\beta }$ (×10^−16^ mol/s)	1.06	2.66	7.14
$J_{ocr}^{\delta }$ (×10^−16^ mol/s)	0.72	1.17	2.43

Cell-to-cell interactions were defined as the number of neighbor cells (either hypoxic or functional) whose membranes were located ≤ 5 *μ*m apart to account for both electrical and paracrine interactions. The number of subnetworks formed between interacting cells (i.e. connected components) was quantified, considering non-viable cells as isolated components. The largest component was defined as the subnetwork containing the highest number of interacting cells.

### Oxygen consumption

Oxygen consumption rates (OCR) for human *α*, *β* and *δ*-cells ($J_{ocr}^{\sigma }$) were estimated based on the experimental measurements of basal OCR per cell by Komatsu et al. [30] in *β*-cell lines (NIT-1 and INS-1) and the *α* TC1 clone 6 line. They showed that the OCR was similar for both cell lines, near 0.005 pmol/min/cell. In addition, Sweet et al. [61] demonstrated a glucose-dependent increase in OCR in human islets (almost a two-fold increase from 3 mM to 20 mM glucose). Similarly, measurements performed by Wang et al. [62] showed a high correlation between OCR and secretion from both non-human and human islets. Based on these experimental measurements, we assumed that changes in OCR are correlated to the changes in the glucose-dependent secretion of glucagon, insulin and somatostatin. That is, a linear increase in OCR as glucose increases for *β* and *δ*-cells, and a U-shaped change in OCR for *α*-cells, as shown by Ramracheya et al. [50] who measured the amount of glucagon, insulin and somatostatin secreted by human islets at glucose concentrations of 1, 6 and 20 mM. OCR values used for the human *α*, *β* and *δ*-cells at 1, 6 and 20 mM glucose are shown in Table 3. As mentioned above, in this model, when cells were identified as non-viable they no longer consumed oxygen. Details about the estimation of oxygen consumption rates are given in S1 Text.

### Glucose uptake

Glucose uptake ($J_{gcr}^{\sigma }$) was implemented based on the experimental data from de Vos et al. [63] who showed that glucose utilization by human islets increases approximately linearly with glucose concentration within the range of 1 and 20 mM. However, since they did not measure the glucose utilization rates for *α*, *β* and *δ*-cells independently and given that *β* and *δ*-cells constitute the majority of cells in human islets (∼ 60% [64]), we used the reported glucose consumption rates by de Vos et al. [63] directly for *β* and *δ*-cells. For *α*-cells, on the other hand, we assumed the maximum glucose utilization reported by de Vos et al. [63] as the consumption rate at low glucose (i.e. 1 mM, when their secretory activity is maximal). At 6 and 20 mM, glucose consumption rates of *α*-cells were estimated following the relative changes in glucagon secretion reported by Ramracheya et al. [50]. Values used are given in Table 3. Non-viable cells were also assumed to cease the consumption of glucose. Details about the estimation of glucose consumption rates can be consulted in S1 Text.

### Modeling encapsulated islets

The reconstructed human islets were enclosed in an additional geometrical domain representing an alginate capsule. The effect of encapsulation on oxygenation and glucose availability within the islets was evaluated simulating a capsule with approximate thickness of 600 *μ*m, the average thickness of the alginate capsules designed experimentally [24, 44, 51, 65]. Alginate was selected as the encapsulating biomaterial for these simulations because it readily allows the diffusion of small molecules such as oxygen, making it a commonly used biomaterial for islet encapsulation [66]. Steady-state simulation of the diffusion of oxygen and glucose through the capsule was simulated using Eqs 1 and 2, using the diffusion coefficients of oxygen and glucose in alginate previously reported ($D_{O_{2}}^{a}=2.5\times 10^{-9}m^{2}/s$ and $D_{G}^{a}=0.6\times^{-9}m^{2}/s$ [30, 42]). The simulations were conducted with initial values of zero for oxygen and glucose concentrations across all domains of the model (capsule and islet). To compare the effect of encapsulation on the availability of oxygen and glucose for islet cells to that observed in the simulations of naked islets, boundary conditions included the same constant oxygen concentrations used for the simulations of non-encapsulated islets (Table 2) and a glucose concentration of 6 mM at the outer capsule boundary following Eqs 3 and 4. On the boundary between the capsule and the islet, a continuity boundary condition was used; that is, $n\cdot (-D_{c}^{a}\nabla \left[C\right]+D_{c}^{t}\nabla \left[C\right])=0$, with *C* indicating either oxygen or glucose concentration, *O*_2_ or *G*, respectively. Oxygen and glucose consumption rates by cells of encapsulated islets was modeled as described before by Eq 3. In order to estimate the time taken for oxygen and glucose to reach the islet due to the diffusive process through the alginate capsule, simulations were performed using the time-dependent diffusion equations (i.e. $\partial \left[C\right]/\partial t=D_{c}^{a}\nabla^{2}\left[C\right]$). Due to the computational cost involved, time-dependent simulations were performed using a time step of 0.5 minutes with total duration of 20 minutes. Schematic diagrams of the models of naked and encapsulated islets, including the definition of boundary conditions can be found in S1 and S2 Figs.

### Computational details

Post-processing and visualizations were performed in Wolfram Mathematica 14 (Wolfram, Inc., Champaign, IL) and Python 3.10. Statistical analyses were performed in GraphPad Prism version 9 (GraphPad Software, Boston, MA, USA). Simulations were performed in a PC with an Intel Core i7 processor (3.8 GHz×16) and 64 GB of RAM memory. Code related to this article, model and architectures files can be consulted in the associated repository (https://github.com/gjfelix/IsletsViability).

## Results and discussion

**Fig 2:**
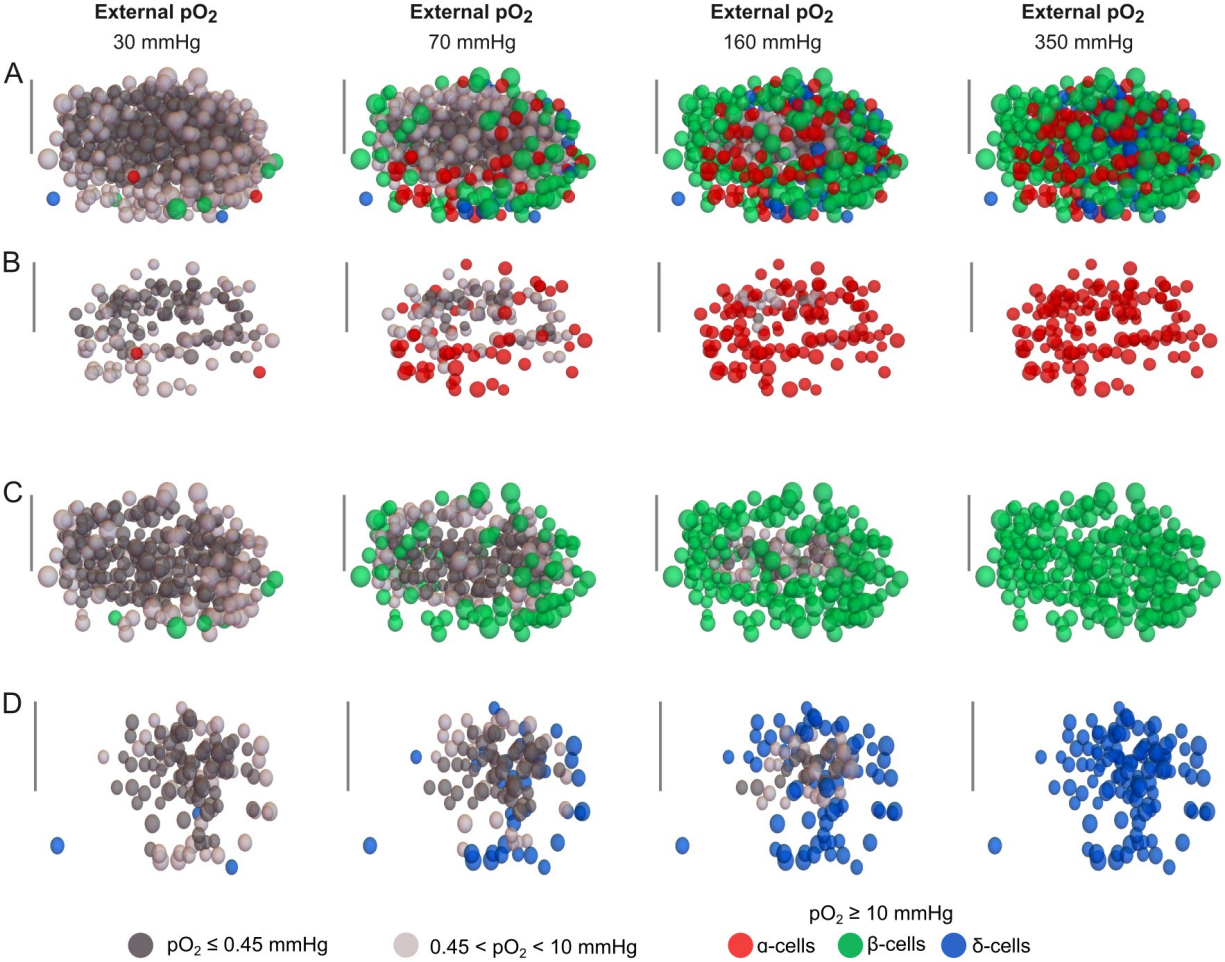
Simulations of oxygenation and cell viability in a human islet at different oxygen partial pressures (*pO*_2_). Oxygenation of islet cells is shown across the entire islet (A) and in the populations of *α*-cells (B), *β*-cells (C) and *δ*-cells (D). The provided simulation corresponds to islet 1 (see Table 1) and was performed at 6 mM glucose concentration. The scale bar represents 50 *μ*m. Non-viable and hypoxic cells are shown in dark and light gray, respectively. Cells within the functional oxygen range as defined in the main text are shown in red (*α*-cells), green (*β*-cells), and blue (*δ*-cells).

The simulation results shown in Fig 2 highlight the capability of the model to estimate the oxygenation level of each cell depending on its identity (*α*, *β* or *δ*) and location within the islet. In contrast to previous models of islet oxygenation that approximate islets as circles (in 2D) or spheres (in 3D) (e.g. [30, 41–43]) without any explicit representation of islet cells, the approach adopted in this work ensures that the diffusion of oxygen and glucose throughout the islet takes into account the morphological details such as the intercellular space, the distribution and size of *α*, *β* and *δ*-cells and their individual consumption of oxygen and glucose. This approach enabled us to perform a quantitative analysis of islet viability with details at the cellular level and to assess the potential impact that the loss of non-viable cells could have on cell-to-cell interactions.

**Fig 3:**
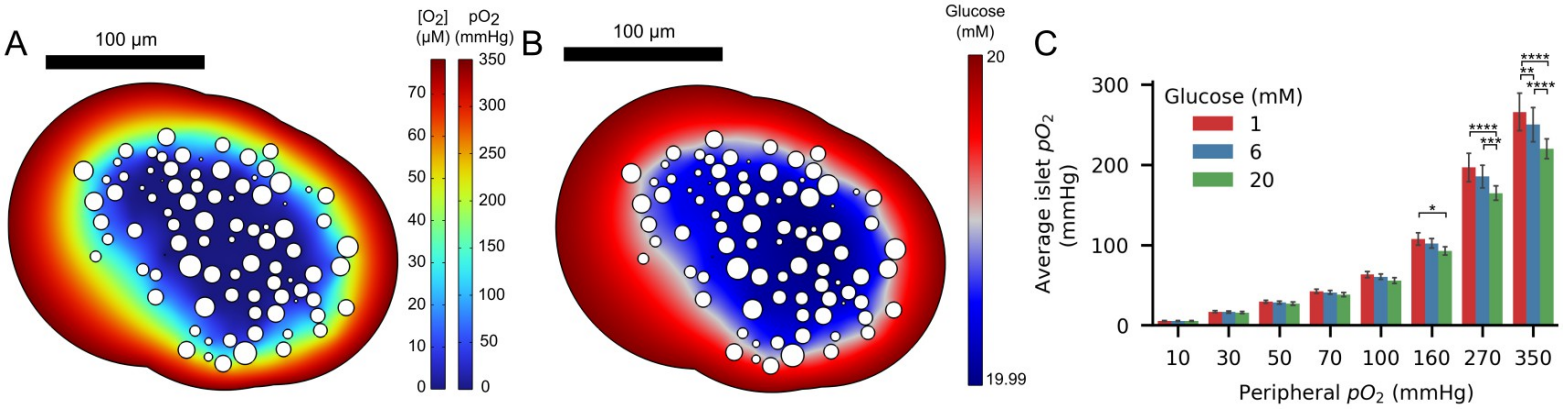
Oxygen and glucose gradients and average oxygen within islets. A and B: Illustration of oxygen and glucose gradients, respectively, formed from the periphery to the islet core as a result of oxygen consumption and glucose uptake (islet 1, 20 mM glucose, peripheral *pO*_2_ = 350 mmHg). C: Average islet *pO*_2_ at 1, 6 and 20 mM glucose across different peripheral *pO*_2_ values. Statistical comparisons between glucose levels for each peripheral oxygen tension were performed using the two-way analysis of variance (ANOVA) followed by Tukey’s test for multiple comparisons (* < 0.1, ** < 0.01, *** < 0.001, **** < 0.0001, non-significant comparisons are not indicated.).

In Fig 3A and 3B, cross-sections of islet 1 are presented, showing the oxygen and glucose gradients predicted by the model during a simulation with 20 mM glucose and peripheral *pO*_2_ of 350 mmHg. It is worth noting that, throughout all the simulations performed, a marked oxygen gradient was observed between the islet periphery and the islet core, while the glucose gradient remained within the micromolar range. This difference can be attributed to the fact that oxygen tension within the islet, when translated to concentration units, lies in the micromolar range (assuming an oxygen solubility in the islet of 2.34×10^−4^ mol/(mmHg ⋅ m^3^) [30]), whereas glucose levels vary in the millimolar range. These results, replicated across all the simulated islets are in agreement with previous studies indicating that glucose consumption and availability have a minimal impact on islet viability [41–43].

Fig 3C shows the average *pO*_2_ within the islets for all the peripheral *pO*_2_ levels and the three glucose concentrations evaluated. As expected, the average islet *pO*_2_ increased with the peripheral *pO*_2_. However, there was also an inverse relationship between the average islet *pO*_2_ and glucose concentration. This effect of glucose concentration on intra-islet *pO*_2_ was more pronounced when the peripheral oxygen tension exceeded 100 mmHg. This result may be related to the percentage of non-viable cells that no longer consume oxygen at lower peripheral oxygen tensions. In contrast, with high peripheral oxygen tensions, a higher proportion of hypoxic and functional cells increases their consumption rates as glucose increases.

**Fig 4:**
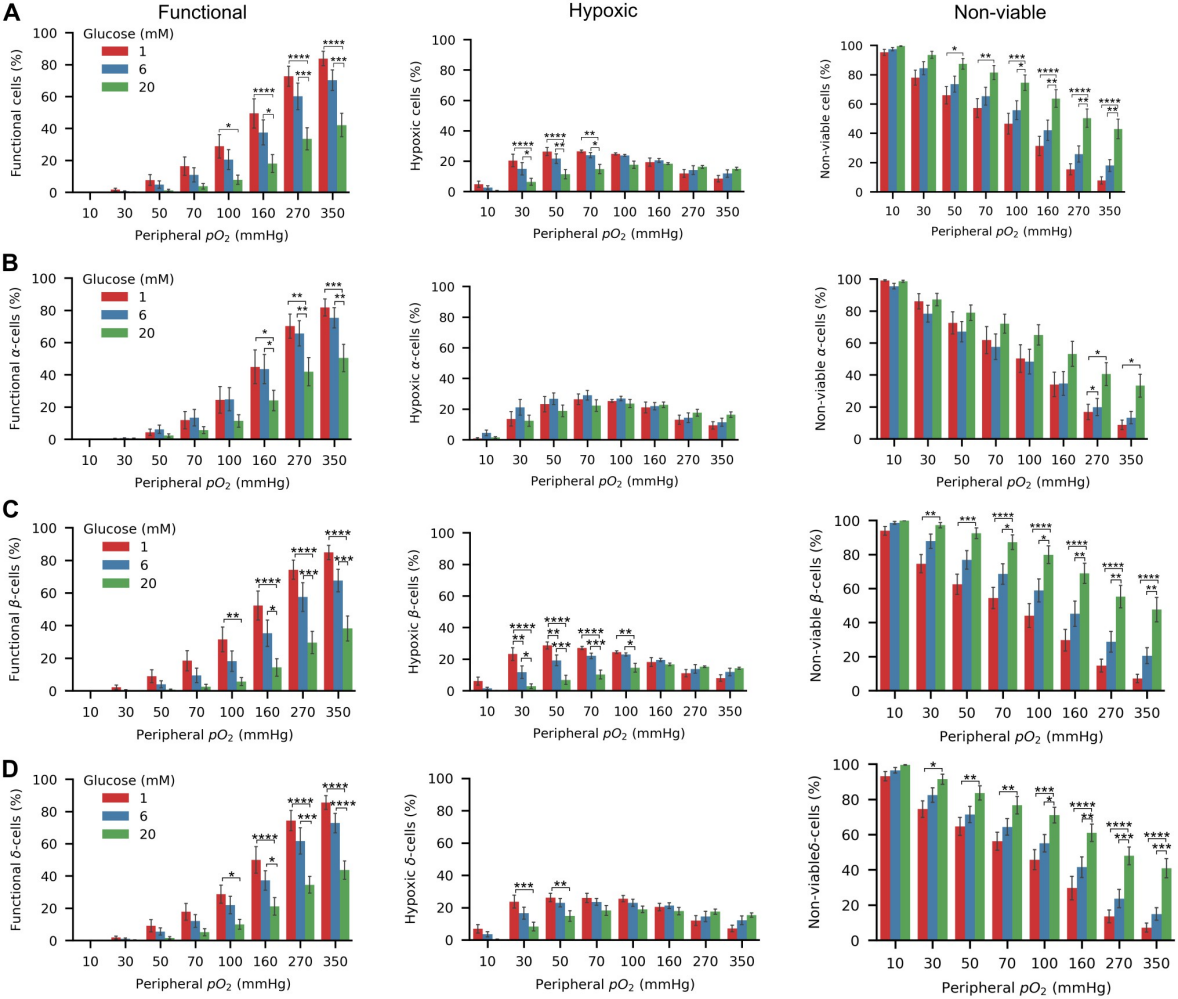
Quantitative analysis of oxygenation of islet cells. Proportions of functional (*pO*_2_ > 10 mmHg, left column), hypoxic (0.45 mmHg ≥ *pO*_2_ ≥ 10 mmHg, middle column) and non-viable cells (*pO*_2_ < 0.45 mmHg, right column). A: All cells. B: *α*-cells. C: *β*-cells. D: *δ*-cells. Statistical comparisons between glucose levels for each peripheral oxygen tension were performed using the two-way analysis of variance (ANOVA) followed by Tukey’s test for multiple comparisons (* < 0.1, ** < 0.01, *** < 0.001, **** < 0.0001, non-significant comparisons are not indicated.

Fig 4A shows how the availability of oxygen within the islets impacts the viability of islet cells at different experimental and transplant conditions, where oxygen levels vary considerably. The subcutaneous space and muscle tissue pose several advantages over other potential transplant sites such as liver, kidney or omentum. These advantages include a relatively simple surgical procedure and the possibility of monitoring and retrieving the implanted islets. Moreover, the extent of skin and muscle tissues increases the available sites for transplantation. Conversely, the main drawback of skin as transplant site is the low availability of oxygen, measured to be 10 mmHg at the superficial layer of the skin [51–53]. At these oxygen levels, the great majority of cells across all the islets simulated were non-viable regardless of glucose concentration (G_1_: 95.2 ± 4.9%, G_6_: 97.4 ± 2.9%, G_20_: 99.5 ± 0.5%), with only a minor percentage (≤5%) reaching the hypoxic state. Increasing the external *pO*_2_ to 30 mmHg, tension measured in muscle tissue and the dermis/subdermis layers of the skin [51, 52, 54, 55], marginally reduced the percentage of non-viable cells (G_1_: 78 ± 12.4%, G_6_: 84.4 ± 10.7%, G_20_: 93.5 ± 5.8%) while increasing the proportion of hypoxic cells (G_1_: 20.4 ± 10.2%, G_6_: 15.0 ± 10.1%, G_20_: 6.3 ± 5.6%) and maintaining a minimal percentage of functional cells (G_1_: 1.6 ± 2.2%, G_6_: 0.7 ± 0.7%, G_20_: 0.2 ± 0.2%).

The liver and portal vein have been the most used transplant sites largely due to their accessibility and the possibility that delivering insulin near the portal vein may be more effective than other infusion sites. On the contrary, as in most potential transplant sites, the main obstacle is the lack of access to oxygen and nutrients, at least until blood flow is restored through the development of a functional vasculature which, according to several reports, could take between one and two weeks after transplantation, although it is also thought that functional maturation of the new vasculature could take several months [67]. Moreover, there is a potential risk of vascular complications such as thrombosis and coagulation, which could compromise both the liver and the transplanted islets. Within the liver, a higher oxygen tension of approximately 50 mmHg has been measured [56]. Replicating these conditions in simulations showed a modest increase in the proportion of functional cells (G_1_: 7.7 ± 8.3% G_6_: 4.8 ± 5.6%, G_20_: 1.3 ± 1.4%), with a higher percentage of hypoxic cells (G_1_: 26.3 ± 6.3%, G_6_: 12.7 ± 7.7%, G_20_: 11.4 ± 7.6%) and the majority of cells still classified as non-viable (G_1_: 66 ± 14.5%, G_6_: 73.5 ± 13.2%, G_20_: 87.3 ± 9%). At the renal cortex, an oxygen partial pressure of 70 mmHg has been measured [59]. Under these conditions, the percentage of cells within the functional oxygenation range increased to 16.3 ± 14.1% for G_1_, 10.9 ± 10.9% for G_6_ and 3.8 ± 4.3% for G_20_. These results can be extended to the bone marrow [52, 57], an alternative transplant site where similar oxygen tensions have been measured [51, 58]. The bone marrow has been proposed as potential site for islet transplantation due to its vast vascularization and extensive distribution throughout the body. However, additional difficulties arising from the immune response might pose a further challenge to graft functionality [34]. Alternative transplant sites have also been investigated, at least in animal models or even in humans in the clinical setting, such as the anterior eye chamber [68], the small intestine [69], the omentum [70] or the spleen [71]. Although not addressed in detail in this work, it is likely that their oxygen tensions fall within the 10–70 mmHg range for transplant sites and the 100–350 mmHg range in culture condition, as evaluated in this work. Consequently, the quantitative analysis performed may be valid for these sites, at least for the period before the development of a functional vasculature.

*In vitro* experiments by Komatsu et al. [60] have demonstrated that increasing the oxygen concentration in the culture environment improves the viability of avascular islets. They measured oxygen partial pressures at varying concentrations: 90 mmHg under 10% oxygen, 160 mmHg under 21% oxygen, 270 mmHg under 35% oxygen and 350 mmHg under 50% oxygen in 1 mL of culture media, respectively. In these conditions (90, 160, 270 and 350 mmHg), the proportion of viable cells of the simulated islets respectively increased to 28.8 ± 18%, 49.4 ± 22.4%, 72.7 ± 15.3% and 83.8 ± 11.1% for G_1_, 20.4 ± 15.3%, 37.5 ± 19.2%, 60.1 ± 20.3% and 70.2 ± 15.6% for G_6_ and 7.9 ± 7.2%, 18.0 ± 13.7%, 33.4 ± 16.9 and 42.1 ± 18% and for G_20_. These results underscores the influence of glucose concentration on the viability of avascular islets. It should be noted that despite the application of high peripheral oxygen tensions a significant proportion of cells remained non-viable. For instance, at G_1_ and a peripheral oxygen tension of 100 mmHg, 46.5 ± 17.3% of cells were non-viable. This percentage decreased to 7.8 ± 6.1% when the peripheral oxygen was increased to 350 mmHg. In contrast, at G_20_ and a peripheral oxygen of 350 mmHg, the percentage of non-viable cells was still considerably high (42.9 ± 16.3%). These results are consistent with those by Komatsu et al. [60] who showed that culturing in hyperoxic conditions at 5 mM glucose contributed to enhance the viability of islet tissue. However, according to our simulations, reducing glucose concentration even further could lead to a substantial increase in cell viability (between 8–13% at G_1_).

In the results described above, we examined the effects of oxygenation on islet cells without differentiating between cell types. However, a closer analysis of the populations of *α*, *β* and *δ*-cells (Fig 4B, 4C and 4D) showed slight differences that are worth highlighting. Specifically, at G_1_, a larger fraction of *α*-cells were non-viable, which can be attributed to their higher oxygen consumption rate compared to *β* and *δ*-cells for all the external oxygen tensions evaluated. Conversely, at G_6_ and G_20_, *β* and *δ*-cell exhibited a greater percentage of non-viable cells when compared to *α*-cells. In fact, for *α*-cells, the highest functionality was observed at 6 mM glucose, which correlates with their minimum oxygen consumption rate. Similarly, for *β* and *δ*-cells, the optimum viability was found at 1 mM glucose, where their oxygen consumption is at its lowest. These results suggest that glucose concentration and oxygen levels in culture should be carefully selected to maximize the viability of islet cells. Our simulations indicate that the distinct populations of *α*, *β*, and *δ*-cells could be affected differently depending on the availability of glucose and oxygen. Moreover, beyond the negative effects on cell viability, hypoxia and necrosis are known to induce pro-inflammatory signals which can exacerbate the immune response, potentially contributing to the failure of the transplanted islets [51, 66].

**Fig 5:**
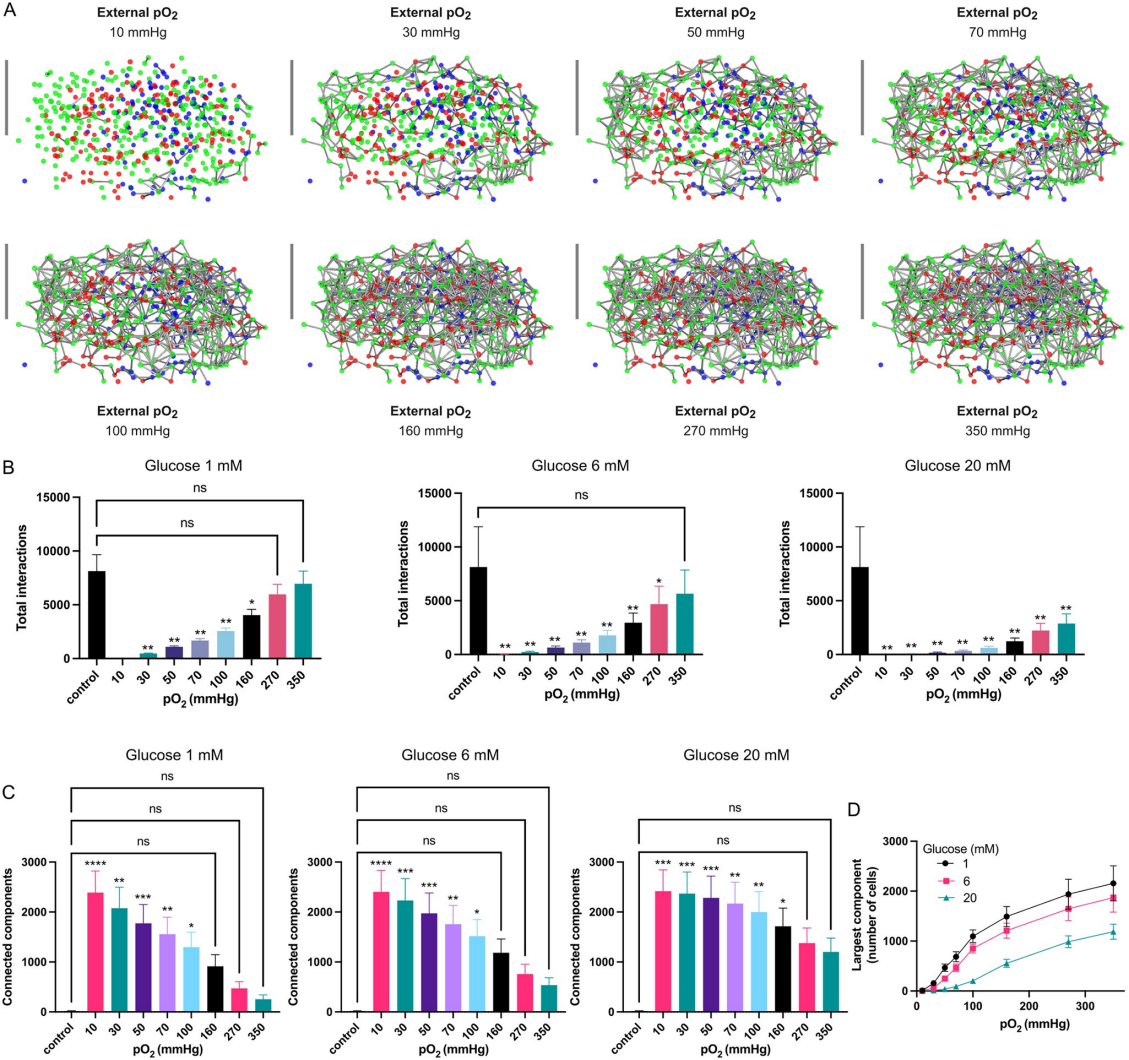
Impact of non-viable cells on cell-to-cell interactions. A: Visualization of cell-to-cell interaction in islet 1. Only the location of the cells is shown (green: *β*-cells, red: *α*-cells, blue: *δ*-cells). Interactions between cells (distance between cell membranes < 5*μ*m) are indicated as gray links. B: Comparison between the average number of total cell-to-cell interactions in control islets (100% viable cells) and avascular islets for different external *pO*_2_ at 1 mM (left), 6 mM (middle) and 20 mM (right) glucose. See S3 Fig for the same comparisons for the different types of interactions between islet cells. C: Connected components formed for different external *pO*_2_ at 1 mM (left), 6 mM (middle) and 20 mM (right) glucose. D: Mean largest component formed depending on glucose concentration and oxygen partial pressure. Significant differences with respect to the control case were determined using the one-way analysis of variance (ANOVA) followed by the post-hoc Dunnett’s test for multiple comparisons (ns: non-significant, **: *p* < 0.0001, *:*p* < 0.01).

As described above, the lack of adequate oxygenation led to cell loss, which inevitably disrupted the potential interactions between islet cells. This effect is illustrated in Fig 5A, where the interactions between cells of islet 1 are shown for different peripheral oxygen tensions at 6 mM glucose. The number of interactions increased with peripheral oxygen, thus highlighting the critical role of adequate oxygenation for the interactions and coordination between *α*, *β* and *δ*-cells. Islet 1 was selected to describe the effects of oxygenation on cell-to-cell interactions in Fig 5A because it was the only one from the six islets simulated that maintained 100% of viable cells, and hence all possible cell-to-cell interactions in hyperoxic conditions (i.e. 270 and 350 mmHg, also shown in Fig 2).

Fig 5B compares the average number of interactions in the simulated islets at varying peripheral oxygen tensions to those in hypothetical control islets, which are assumed to have 100% viability and cell-to-cell interactions. For the three glucose concentrations simulated, the average number of interactions increased with higher peripheral oxygen tensions. However, at G_20_ the reduction in cell-to-cell interactions was more pronounced as a result of the higher oxygen consumption by *β* and *δ*-cells which led to a lower availability of oxygen within the islets and the consequent loss of cells. For the control islets, the average number of interactions was 8132 ± 3745. In comparison, at G_1_, rising the peripheral *pO*_2_ from 10 to 350 mmHg led to an increase in the average number of interactions from 30.5 ± 10.2 to 6959 ± 2861, which represents 0.38% and 85.6% of the control, respectively. At G_20_, these figures were considerably lower, decreasing to 1.3 ± 1.5 and 2875 ± 902.5 (corresponding to 0.02% and 35.4% of the control case, respectively). For G_6_, only the highest peripheral oxygen tension (350 mmHg) allowed cell-to-cell interactions to approach levels observed in the control case. Notably, at G_20_, none of the peripheral oxygen tensions evaluated in our simulations enabled the average number of cell-to-cell interactions to reach values comparable to the control case, as can be seen in the right panel of Fig 5B. The loss of cell-to-cell interactions resulted in the segregation of the islets, as shown in Fig 5C, measured by the number of connected components (or subnetworks) at different glucose concentrations and oxygen partial pressures. Control islets (i.e. assuming 100% viability) formed an average of 18.00 ± 14.52 subnetworks, with the largest component containing 2391.83 ± 1041.97 cells. Compared to control islets, the number of subnetworks increased significantly at peripheral oxygen levels between 10 and 100 mmHg for G_1_ and G_6_, and between 10 and 160 mmHg for G_20_. However, within these ranges, the number of connected components decreased as peripheral oxygen increased (Fig 5C). At higher *pO*_2_ levels (i.e. > 100 mmHg for G_1_ and G_6_ and > 160 mmHg for G_20_), the number of subnetworks did not differ significantly from control islets, although a high number of connected components persisted (Fig 5C). This decrease in connected components correlated with an increase in their size, as shown by the size of the largest components in Fig 5D. Functionally, these results suggest that the loss of cell-to-cell interactions (i.e. connectivity) produced by lack of oxygen could impair islet cell coordination, even under hyperoxic conditions.

Specific cell-to-cell interactions (*α* − *α*, *β* − *β*, *δ* − *δ*, *α* − *β*, *α* − *δ* and *β* − *δ*), were also quantified. At G_1_, practically all the specific interactions followed the behavior shown in Fig 5B; that is, when the peripheral oxygen tension was either 270 or 350 mmHg, the specific interactions between islet cells were comparable with the control case. At G_6_, however, while the *α*-*α*, *δ*-*δ*, and *α*-*δ* interactions reached similar levels to the control case for peripheral oxygen tensions greater than 270 mmHg, *α*-*β* interactions were only comparable to the control case at 350 mmHg. Interestingly, even at higher peripheral oxygen tensions, *β*-*β* and *β*-*δ* interactions failed to reach levels similar to the control case. At G_20_, interactions between the three types of cells were severely disrupted. S3 Fig provides a quantitative analysis of the specific cell-to-cell interactions.

**Fig 6:**
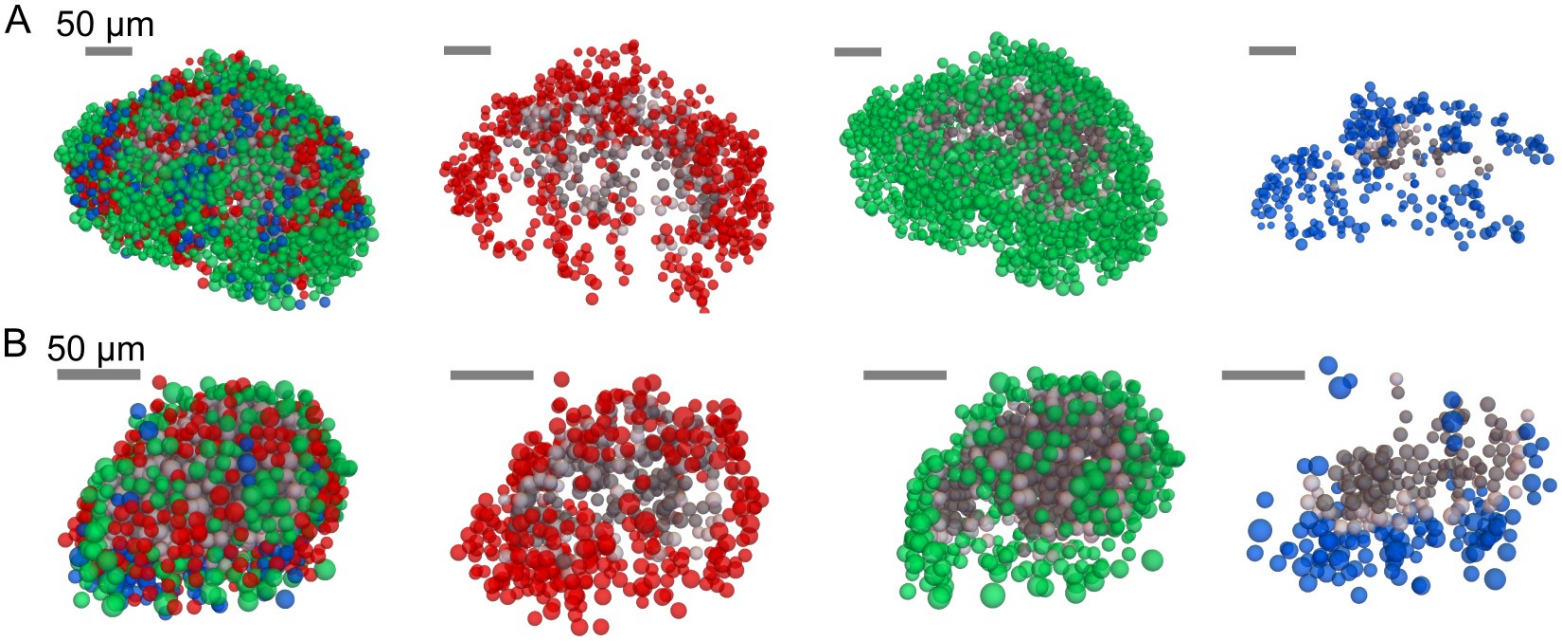
Simulations of oxygenation of islet cells and cell viability in human islets under hyperoxic conditions (350 mmHg). A. Islet 4 (3516 cells) with 1 mM glucose. B. Islet 5 (2084 cells) at 20mM glucose. In A and B, oxygenation of islet cells is shown for the whole islets (first column) and populations of *α* (second column), *β* (third column) and *δ*-cells (fourth column).

It is well established that small islets are more suitable for transplantation as experimental evidence and computational simulations have shown that small islets are more likely to remain viable [30, 49, 72–75]. A qualitative analysis of our simulations results, focusing on islets encompassing the full range of islet sizes (islet 1: 583 cells, islet 5: 2084 cells and islet 4: 3516 cells, see Figs 2 and 6) indicated that only for islet 1, the smallest of the islets simulated, all cells remained in the functional range at peripheral oxygen tensions of 270 and 350 mmHg (see the last column of Fig 2). These oxygen tensions are much higher than those measured in potential transplant sites and are only achievable in hyperoxic experimental conditions [60] (see Table 2). In contrast, for larger islets such as islets 4 and 5, shown in Fig 6, a considerable proportion of cells were identified as non-viable or hypoxic even at hyperoxic *in vitro* conditions.

**Fig 7:**
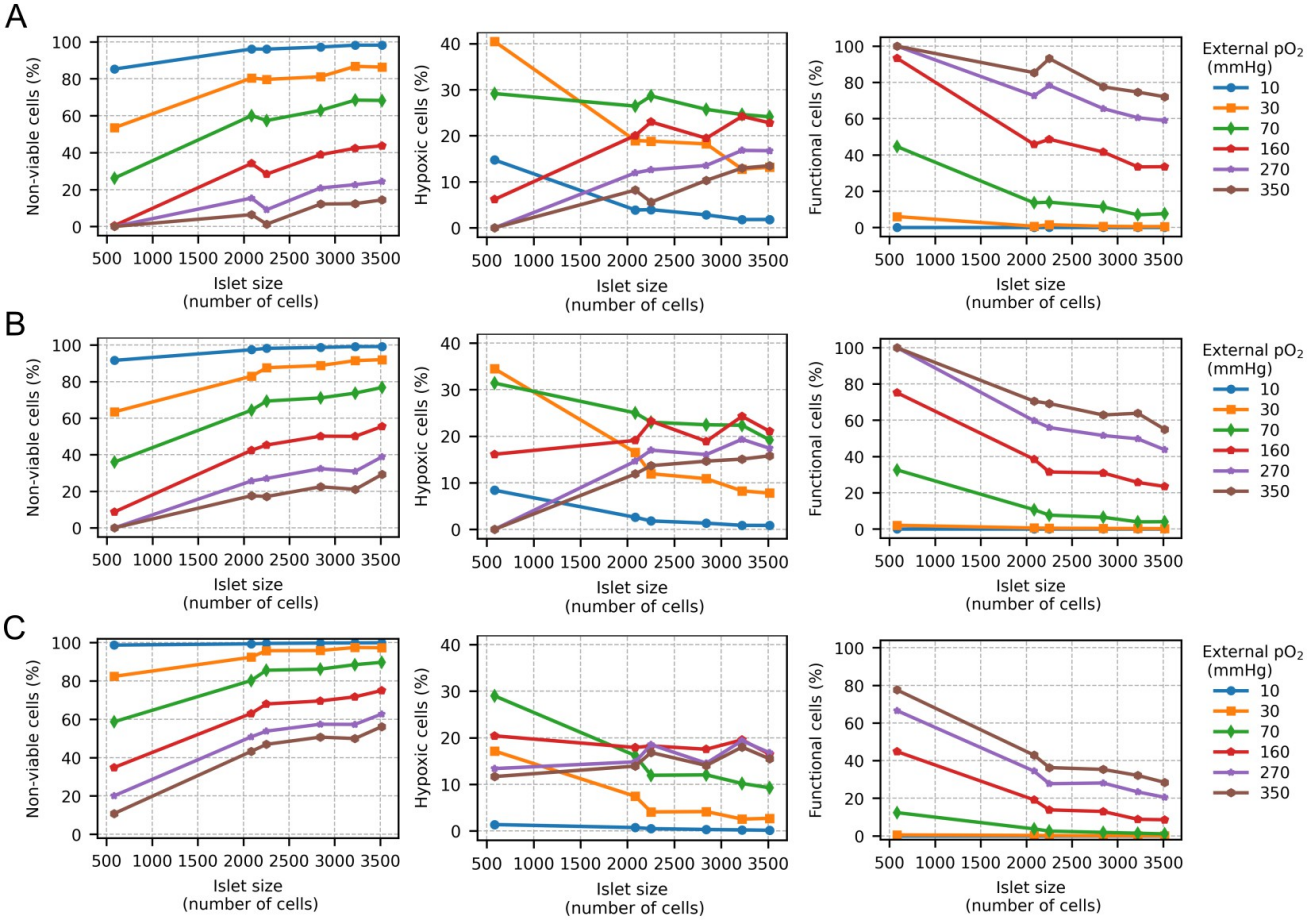
Effect of islet size, denoted as number of cells, on viability of islet cells. A-C. Nonviable (left), hypoxic (middle) and functional cells (right) for different external *pO*_2_ with 1, 6 and 20 mM glucose (A, B and C, respectively). Note the different scale in the plots of the percentage of hypoxic cells (middle column).

The effect of islet size, denoted as the number of cells, on the proportion of non-viable, hypoxic and functional cells, due to the availability of glucose and oxygen, is described in Fig 7A, 7B and 7C for G_1_, G_6_ and G_20_, respectively, and variable peripheral oxygen tensions. For the three glucose concentrations, an increase in the percentage of non-viable cells was observed with increasing islet size, although this effect was more pronounced at G_20_ compared to G_1_. For example, at a peripheral *pO*_2_ of 350 mmHg and G_1_, the largest islet (3516 cells) had 14.4% non-viable cells, whereas at G_20_ it showed 56% of non-viable cells. Conversely, the percentage of functional cells was inversely correlated to both islet size and glucose concentration. An exception to this was the peripheral oxygen tension of 10 mmHg, where no functional cells were found at any glucose concentration, as shown in the right column of Fig 7A, 7B and 7C. The smallest islet (583 cells) at hyperoxic *in vitro* conditions (> 160 mmHg) and G_1_ maintained over 90% of functional cells (93.3% for 160 mmHg and 100% for 270 and 350 mmHg). These percentages dropped to 72.1% (350 mmHg), 59.3% (270 mmHg) and 34% (160 mmHg) for the largest islet (3516 cells) even at 1 mM glucose. Increasing glucose to 20 mM produced a decrease of 20–30% in the percentage of functional cells regardless of islet size. In G_6_, similar trends were observed although to a lesser extent due to the reduced oxygen consumption by islet cells in comparison to that at 20 mM glucose. The percentage of hypoxic cells, shown in the middle column of Fig 7A, 7B and 7C, varied depending on the peripheral *pO*_2_. At 10, 30 and 70 mmHg, the percentage of hypoxic cells was inversely correlated to islet size for all glucose concentrations. On the other hand, under hyperoxic *in vitro* conditions (peripheral *pO*_2_ > 160 mmHg), the percentage of hypoxic cells increased with islet size, although this effect was less evident for G_20_. At first, it could seem counterintuitive that the percentage of hypoxic cells at 10 mmHg is lower than at higher peripheral oxygen tensions. However, at 10 mmHg, most cells are non-viable, with only a few within the hypoxic range. In contrast, at 350 mmHg most cells are within the functional range even for larger islets, with more cells in the hypoxic range and only a few in the non-viable range.

**Fig 8:**
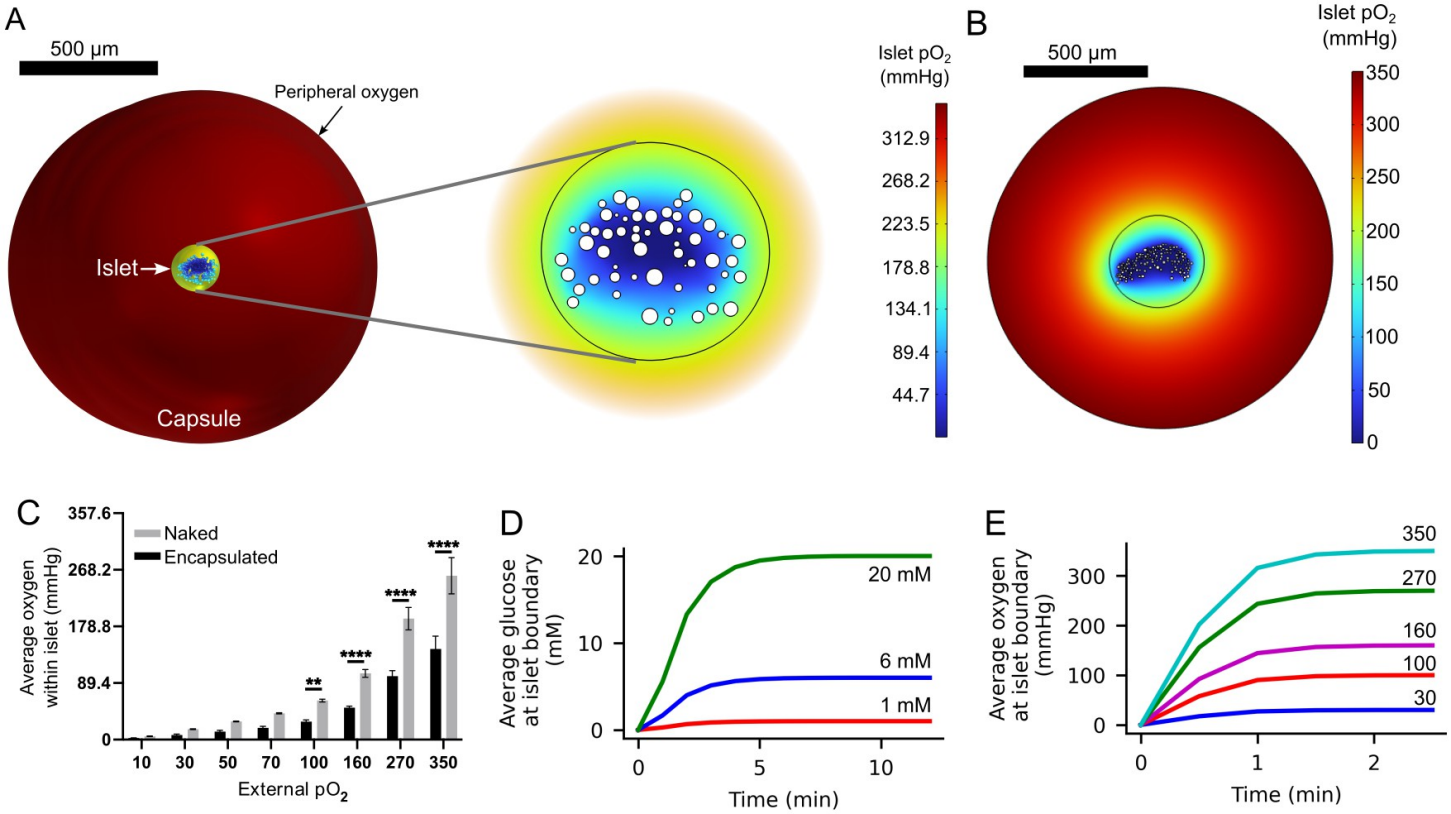
Oxygen gradient in an encapsulated human islet. A. An example of an encapsulated islet (islet 1) with a capsule of 600 *μ*m thickness (left). The inset (right) shows a closer view of the islet. Both panels share the same color legend. B. Oxygen gradient formed between the capsule boundary and the core of islet 4. C. Effect of encapsulation on the average islet oxygen for different peripheral oxygen tensions. Statistical comparisons between encapsulated and naked islets for each peripheral oxygen oncentration were performed using the two-way analysis of variance (ANOVA) followed by Tukey’s test for multiple comparisons (** < 0.01, **** < 0.0001, non-significant comparisons are not indicated). D and E. Encapsulation produced a delay in the formation of glucose (D) and oxygen (E) gradients.

Although this article mainly focuses on simulating the diffusion of glucose and oxygen throughout avascular islets using realistic architectures, the modeling approach here adopted can be extended to further simulate other scenarios commonly evaluated using computational models such as islet encapsulation. As a proof of concept, steady state simulations of encapsulated islets were performed (see Fig 8A and 8B). In these simulations, capsules with a thickness of 600 *μm* (the average thickness of the alginate capsules used experimentally [24, 44, 51, 65]), were added to the models of islets 1, 2 and 4.

In order to determine the effect of encapsulation as an additional physical barrier on the availability of oxygen and the viability of the islet cells, the simulated encapsulated islets were subject to a glucose concentration of 6 mM and the same oxygen concentrations as the simulated naked islets (see Table 2). These simulations indicated that encapsulation had a deleterious effect on the availability of oxygen within the islet, as shown in Fig 8C, especially when culture oxygen was high (> 100 mmHg). Particularlly, encapsulation reduced the average oxygen within the islet by 51.5 ± 4.95%. Additionally, preliminary time-dependent simulations evidenced that encapsulation delayed the establishment of glucose and oxygen gradients. An example of this delay is shown in Fig 8D and 8E, where the average glucose concentration and oxygen tension in the islet boundary are shown for different peripheral *pO*_2_. As expected, due to the differences in the diffusion coefficients of oxygen and glucose in the capsule, average glucose at the islet boundary reached the steady state in approximately 10 minutes regardless of the external glucose concentration (Fig 8E). In contrast, the average oxygen tension in the islet boundary reached the steady state in about 2 minutes (Fig 8D). These delays could pronounce the negative effects of isolation of the pancreatic islets by inducing temporary hypoxia due to the additional physical barrier to nutrients and oxygen, potentially leading to irreversible damage of islet cells even if revascularization occurs at a later stage [76, 77]. In addition to encapsulation, the modeling approach proposed here is suitable to simulate other scenarios of interest. For instance, it is known that the implantation of encapsulated islets can lead to the formation of a thrombus or a fibrotic capsule over the encapsulating biomaterial [45, 78] as a result of the immune response to the implant, which involves the foreign body response and the recruitment of immune cells to form the fibrotic capsule [79, 80]. To model this scenario, two additional factors would have to be considered: the domain representing either the thrombus or the fibrotic capsule, and the increased consumption of oxygen due to the recruited immune cells, which would further decrease the oxygen tension at the implant site [66].

**Fig 9:**
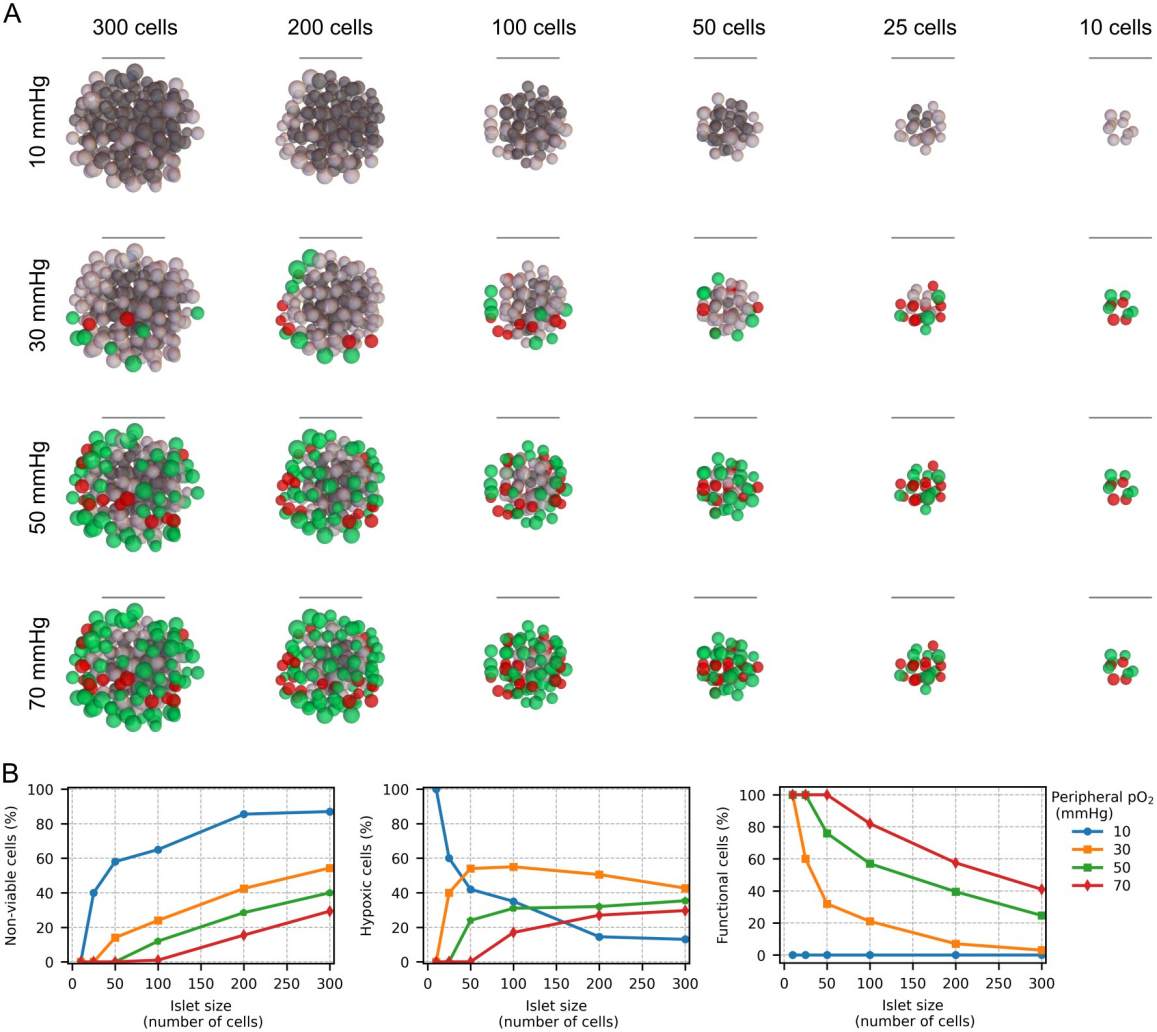
Viability of microislets in transplant oxygenation conditions. A. Simulations of oxygenation of microislets of varying sizes (10 to 300 cells) with peripheral oxygen tensions ranging from 10 to 70 mmHg. B. Percentage of non-viable (left), hypoxic (middle) and functional cells (right) in the microislets shown in A.

The results of the simulations presented above indicate that human islets with fewer than 600 cells can remain fully viable only under hyperoxic culture conditions (> 270 mmHg). In contrast, even the smallest islet showed a high percentage of non-viable cells under transplantation oxygenation conditions (10–70 mmHg). Furthermore, even if the immune response against the transplanted islets could be neutralized by encapsulation, our simulations suggest that the oxygen availability for encapsulated islets would be further compromised. Based on these findings, it is evident that transplanted islets must be considerably smaller than the reconstructed islets used in this study. To estimate the appropriate size of avascular islets for transplantation considering different oxygenation transplant conditions, we simulated islets ranging from 10 to 300 cells, in the following referred to as microislets (Fig 9A). As depicted in Fig 9B, at 10 mmHg, only extremely small microislets (10 cells) showed all cells in the hypoxic range. Larger islets at 10 mmHg showed both non-viable and hypoxic cells with a growing percentage of non-viable cells as the size of the microislets increased. At higher peripheral oxygen tensions (> 30 mmHg), microislets with 10 cells were capable of maintaining all cells in the functional range. For microislets of 25 cells, only peripheral oxygen tensions of 50 and 70 mmHg maintained all cells within the functional range while at 30 mmHg some hypoxic cells were observed (without non-viable cells). At 30 mmHg, microislets bigger than 30 cells showed a considerable percentage of non-viable cells. Only at 70 mmHg, microislets with 50 and 100 cells could remain within the hypoxic or functional range, although a minor percentage of non-viable cells were found. In microislets bigger than 100 cells, at all peripheral oxygen tensions, there was a non-negligible percentage of non-viable cells. Based on these results, if the aim is to preserve the transplanted cells in the functional oxygen range, our simulations suggest that islets larger than 100 cells should be avoided in transplant sites with oxygen tensions near 70 mmHg (e.g. renal cortex). At the intestine, liver or bone marrow, where the oxygen tension lies around 50 mmHg, microislets composed of maximum 50 cells appear to be the most suitable for transplantation. In the subcutaneous space or in muscle tissue, microislets with fewer than 25 cells could be used while, at the superficial skin, only extremely small microislets (10 cells) could be used for transplantation according to our simulations.

From the computational viewpoint, the model presented in this work is more computationally expensive than previous models of avascular islets used in recent years, which, as previously mentioned, are based on simplified geometries, and therefore neglect relevant morphological details at both the islet and the cellular level. Despite this, the modeling approach proposed here has the following advantages:

- It is based on realistic islet architectures reconstructed from experimental data.
- It distinguishes between different cell populations (*α*, *β* and *δ*).
- It provides a detailed description of the oxygenation and viability of each cell within the islet.
- It allows variable oxygen consumption rates depending on the glucose concentration and cell type.
- It offers quantitative information of the impact oxygenation on the connectivity between islet cells.
- It is extendable to simulate other scenarios of interest (e.g., encapsulation, fibrotic capsule, etc.) and can be used for islets from different species.

Conversely, this level of detail also poses some limitations. For instance, detailed time-dependent simulations are still not possible due to the spatial complexity involved which requires an extremely fine spatial discretization. This spatial complexity compromises the possibility of performing simulations with an equally fine temporal discretization. For this reason, simulating dynamic processes such as hormone secretion or varying glucose concentrations and other valuable scenarios remains elusive with the available computational resources. Nevertheless, we are convinced that the model here presented constitutes a step forward in the field of computational models of pancreatic islets. Consequently, future models that incorporate detailed biophysical properties at the cellular level, including the electrophysiological and secretory functions of *α*, *β*, and *δ*-cells, as well as cell-to-cell communication signals, will provide the opportunity to develop comprehensive computational models to investigate the functional implications of hypoxia on the functioning of islet cells, and in understanding how the loss of cell mass affects electrical and paracrine communication within the islets.

## Conclusions

In this work, we presented a detailed model of avascular human islets aiming to contribute to improve culture and transplant conditions by estimating the optimal glucose concentration and culture oxygen tension depending on islet morphology, cell number, composition, cell type, size and location. Our simulations suggest that culturing avascular islets at high glucose concentrations could have a significant negative effect on the viability of islet cells in comparison to cultures at low glucose concentrations. We also showed that at oxygen tensions measured at potential transplant sites (10–70 mmHg) even for the smallest islet simulated (583 cells) the majority of cells could be subject to severe hypoxia. According to our simulations, small islets could be maintained entirely functional at hyperoxic experimental conditions (*pO*_2_ > 270 mmHg). The viability of bigger islets (> 2000 cells), on other hand, was only maintained over 70% for the lowest glucose concentration and high external oxygen tensions (> 270 mmHg). Following these results, potential cell-to-cell interactions were only comparable to the hypothetical control case with fully functional cells and cell-to-cell interactions at high peripheral oxygen tensions (> 270 mmHg) and low glucose concentrations, while at high glucose (20 mM) cell-to-cell interactions were considerably disrupted, even at hyperoxic conditions. Although it was already acknowledged that small islets were more likely to maintain a higher percentage of viable cells, we have shown that in addition to islet size, glucose concentration and peripheral oxygen tensions must be carefully selected in order to maximize the viability and functionality of avascular islets either for *in vitro* studies or transplantation. Furthermore, our simulations indicate that microislets composed of fewer than 100 cells could be more suitable for transplantation. In addition to evaluating the viability of islet cells in different culture and transplant conditions, the modeling approach here proposed has potential applications such as predicting the impact of encapsulation devices on the viability of different populations of cells or identifying the effect of transplant complications such as thrombosis or fibrosis on the oxygenation of transplanted islets.

## Supporting information

S1 Fig Schematic diagram of the models of naked islets. (TIFF)

S2 Fig Schematic diagram of the models of encapsulated islets. (TIFF)

S3 Fig Quantitative analysis of specific cell-to-cell interactions for 1 mM glucose (A), 6 mM glucose (B) and 20 mM glucose (C). (TIFF)

S1 Text Phenomenological models of oxygen consumption rates and glucose uptake by human *α*, *β* and *δ*-cells. (PDF)
