# Supplementary figures and images for: Impact of oxygen and glucose availability on the viability and connectivity of islet cells: A computational study of reconstructed avascular human islets
Source: PLoS Comput Biol. 2024 Aug 13;20(8):e1012357. doi: 10.1371/journal.pcbi.1012357 (PMC11343470; doi:10.1371/journal.pcbi.1012357)

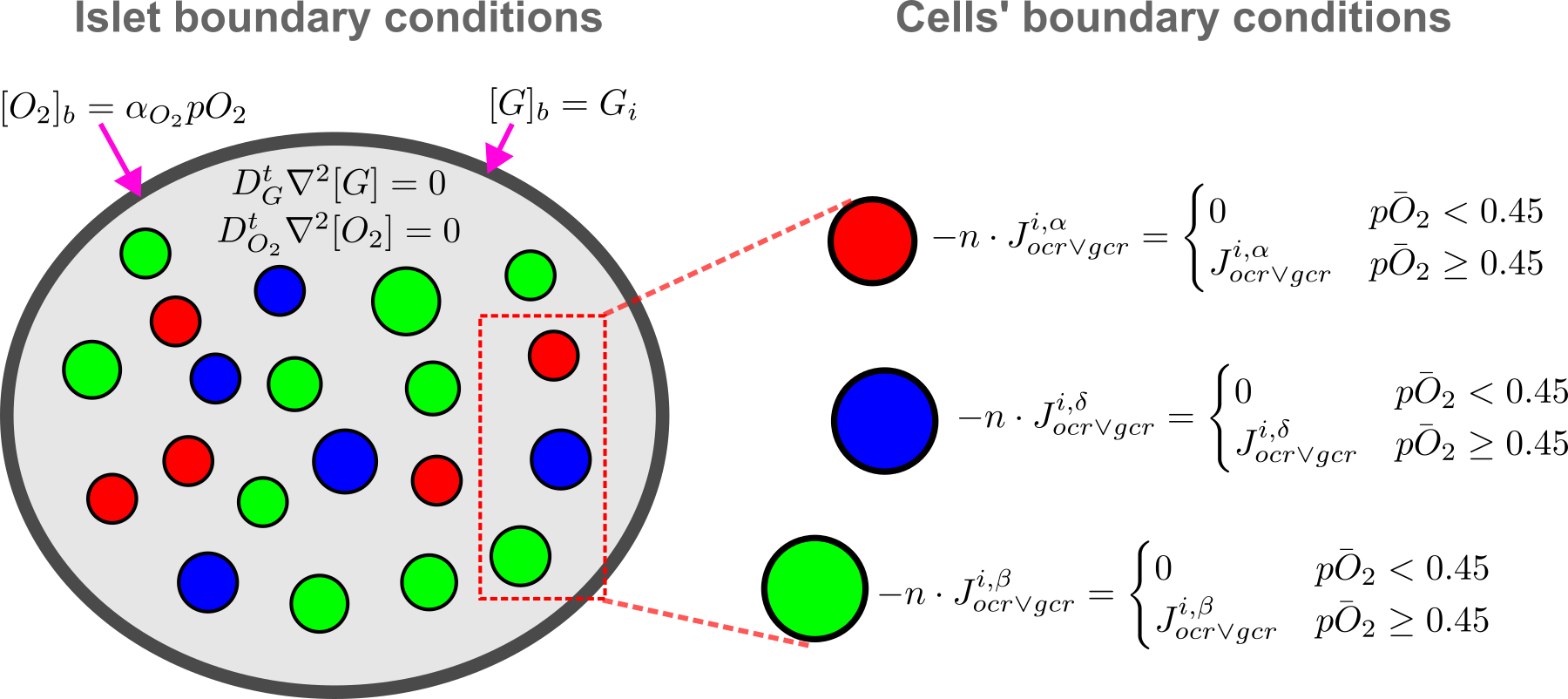

Supplement: S1 Fig — (TIFF) [file pcbi.1012357.s001.tiff]

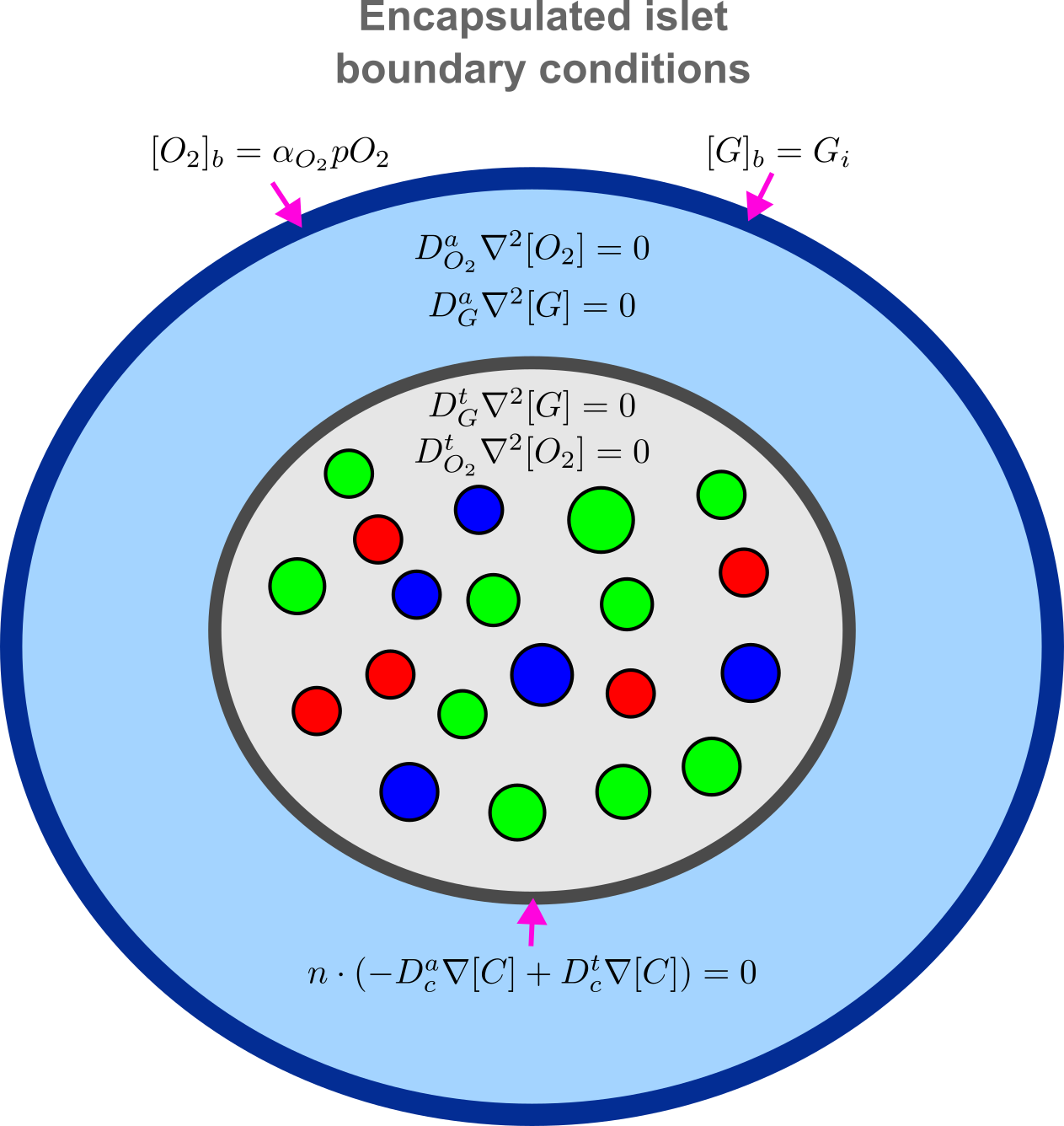

Supplement: S2 Fig — (TIFF) [file pcbi.1012357.s002.tiff]

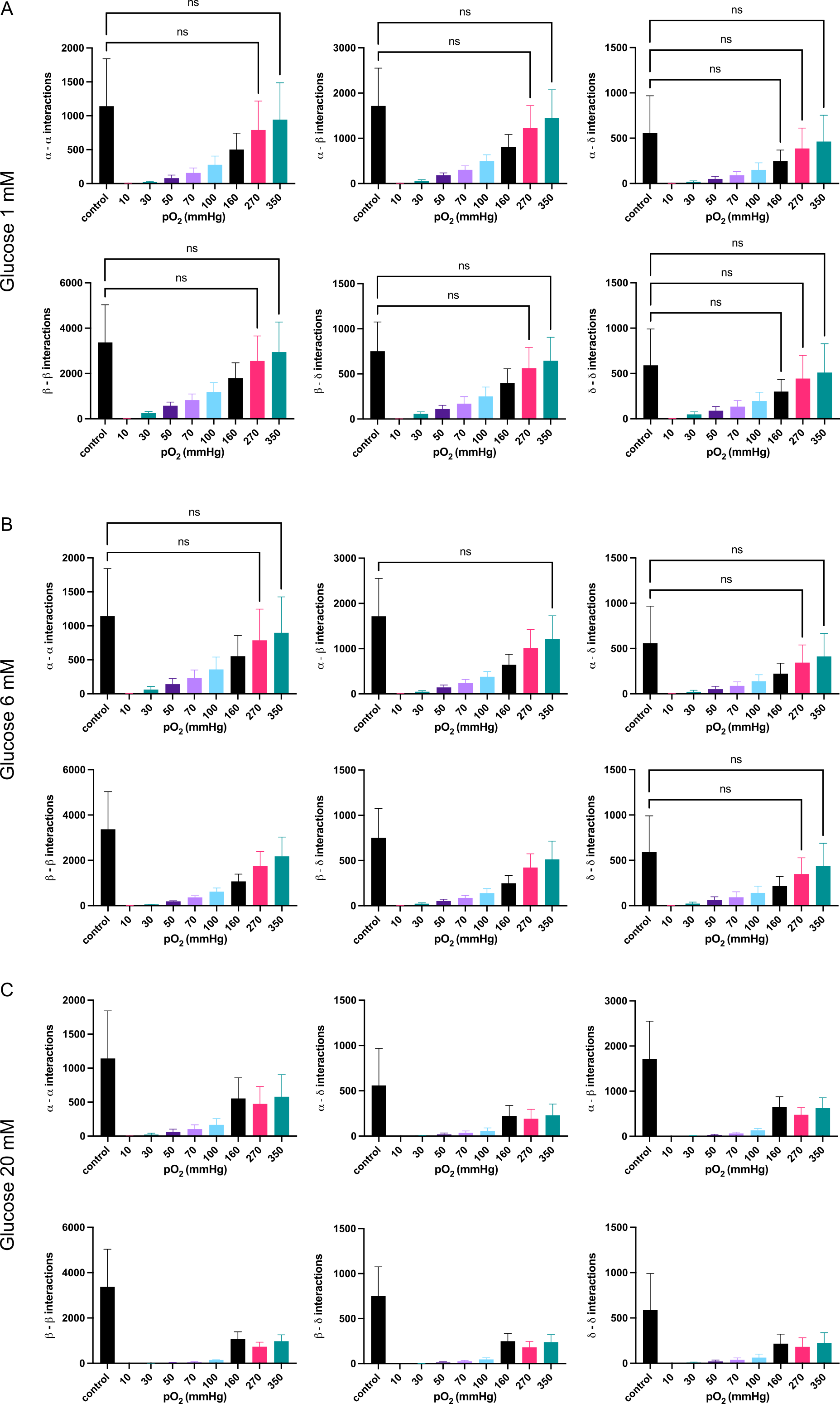

Supplement: S3 Fig — (TIFF) [file pcbi.1012357.s003.tiff]
