# Supplementary material for: Impact of oxygen and glucose availability on the viability and connectivity of islet cells: A computational study of reconstructed avascular human islets
Source: PLoS Comput Biol. 2024 Aug 13;20(8):e1012357. doi: 10.1371/journal.pcbi.1012357 (PMC11343470; doi:10.1371/journal.pcbi.1012357)
Supplement: S1 Text — (PDF) [file pcbi.1012357.s004.pdf]

S1 Text. Phenomenological models of oxygen consumption rates and glucose uptake by human α, β and δ-cells

Oxygen consumption rates

Mathematically, OCR for the viable human α, β and δ-cells in the glucose range between 1 and 20 mM was defined by phenomenological models as:

$$J_{ocr}^{\alpha} = 2.4 \times 10^{-16} - 3.6 \times 10^{-17}\bar{G}_i + 1.5 \times 10^{-18}\bar{G}_i^2 \quad (1)$$

$$J_{ocr}^{\beta} = 7.4 \times 10^{-17} + 3.2 \times 10^{-17}\bar{G}_i \quad (2)$$

$$J_{ocr}^{\delta} = 6.3 \times 10^{-17} + 9.0 \times 10^{18}\bar{G}_i \quad (3)$$

in units of mol/s with $\bar{G}_i$, the average glucose concentration at the surface of cell i in mM. Fig A (left) shows these relationships for the three types of cells. OCR was implemented in the model as a negative surface flux for each cell of the reconstructed islet taking into account the surface area of that is, $J_{ocr,i}^{\sigma} = -J_{ocr}^{\sigma}/A_i$ in units of mol/m^2/s, where σ indicates the type of cell (α, β, δ) and A_i is the surface area of the cell membrane.

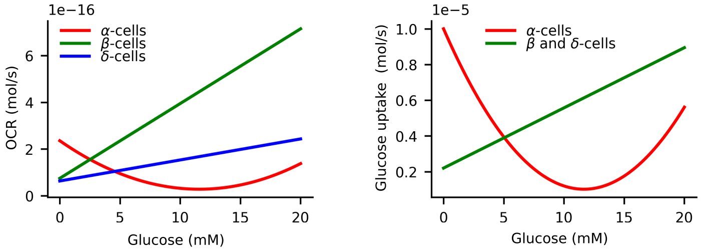

Figure A: **Phenomenological models of the oxygen consumption rates (OCR) and glucose uptake for α, β and δ-cells.** Left: OCR for α, β and δ-cells (Eqs 1-3). Right: Glucose uptake as described mathematically by Eqs 4 and 5.

Glucose uptake

The glucose uptake for α, β and δ-cells as a function of glucose is shown in Fig A (right). Similarly to the OCR, glucose uptake was implemented as a negative surface flux given by $J^{\sigma}_{gcr,i} = -J^{\sigma}_{gcr}/A_i)$, with σ indicating the type of cell, A_i the surface area of the cell membrane and J^{σ}_{gcr} described mathematically (in units of pmol/s) as:

$$J^{\alpha}_{grc} = 1.0 \times 10^{-5} - 1.54 \times 10^{-6}\bar{G}_i + 6.6 \times 10^{-8}\bar{G}_i^2 \quad (4)$$

$$J^{\beta}_{grc} = J^{\delta}_{grc} = 2.2 \times 10^{-6} + 3.4 \times 10^{-7}\bar{G}_i \quad (5)$$
